# The Form of a Conditioned Stimulus Can Influence the Degree to Which It Acquires Incentive Motivational Properties

**DOI:** 10.1371/journal.pone.0098163

**Published:** 2014-06-06

**Authors:** Paul J. Meyer, Elizabeth S. Cogan, Terry E. Robinson

**Affiliations:** Department of Psychology, University of Michigan, Ann Arbor, Michigan, United States of America; Centre for Addiction and Mental Health, Canada

## Abstract

There is considerable individual variation in the extent to which food- and drug-associated cues (conditioned stimuli, CSs) acquire incentive salience, as indicated by whether they elicit approach towards them, and/or act as conditioned reinforcers. Here we asked whether this variation is influenced by properties of the CS itself. In rats, we assessed both the attractiveness and conditioned reinforcing properties of two CSs: a manipulable lever CS versus an auditory (tone) CS. There was considerable individual variation in the extent to which a lever CS acquired incentive motivational properties, as indicated by whether it became attractive (evoked a sign-tracking or goal-tracking conditioned response) or acted as a conditioned reinforcer. However, with a tone CS all rats learned a goal-tracking response, and the tone CS was an equally effective conditioned reinforcer in sign-trackers and goal-trackers. Even when presented in compound (a lever-tone CS), the two elements of the compound differentially acquired motivational properties. In contrast, amphetamine and stress potentiated the conditioned reinforcing properties of both visual and auditory CSs similarly in rats that primarily sign-tracked or goal-tracked. We conclude that variation in the to the ability of CSs to acquire incentive salience, and thus their ability to act as incentive stimuli capable of motivating behavior, is determined in part by properties of the CS itself.

## Introduction

Conditioned stimuli (CSs, or “cues”) associated with rewards (unconditioned stimuli, USs) can evoke many different conditioned responses (CRs). The form of a given CR is determined in part by the properties of the US, but many other factors also play a role, including the nature of the CS itself [1], and of particular interest here, whether a CS acquires incentive motivational properties [2]–[5]. If a CS is attributed with incentive salience it can act as an *incentive stimulus*, capable of evoking powerful emotional and motivational states. Incentive stimuli have three fundamental properties: (1) they become attractive, biasing attention towards them and eliciting approach into close proximity with them; (2) they are themselves desired, in the sense that animals will learn new instrumental actions to get them (i.e., they act as conditioned reinforcers); and (3) they arouse conditioned motivational states capable of instigating reward-seeking behavior or energizing ongoing seeking behavior [6]–[8].

There is, however, considerable individual variation in the extent to which CSs are attributed with incentive salience ([3], [4], [9]–[10], for recent reviews see [5], [11]–[12]). For example, if rats are trained using a standard Pavlovian Conditioned Approach (PCA; ‘autoshaping’) procedure, in which a CS (an illuminated lever) is paired with food delivery, they may develop one of two distinct CRs. As first described by Boakes [12], some rats (“sign-trackers”; STs) show CS-directed responses that involve approach to and vigorous nibbling, sniffing and biting the lever CS itself, whereas other rats (“goal-trackers”; GTs) initially glance at the lever, then approach the location of the food delivery and nibble and sniff in the food cup, prior to delivery of the food pellet [13]. The remainder of the rats (“intermediates”; IN) vacillate between these two responses. Thus, ST and GT CRs are very similar in both the initial approach component, and in the apparently “consummatory-like” actions displayed towards the lever or food cup, respectively – but they are directed at different targets [14], [15]. Importantly, the behavior of STs and GTs during PCA training predicts their behavior on other tests designed to determine if a CS has acquired incentive stimulus properties. For example, a lever or light CS paired with either food or cocaine is also a more effective conditioned reinforcer and more effective in producing conditioned motivation (defined as instigating reward-seeking behavior), in STs than in GTs [16]–[24]. Thus, a localizable CS (lever or light) associated with either food or cocaine acquires the properties of an incentive stimulus to a greater degree in STs than GTs (for reviews see [4], [5]).

The purpose of the studies reported here was two-fold: 1) to determine whether auditory CSs differentially acquire incentive value in STs and GTs, and 2) to determine whether amphetamine and stress potentiate the reinforcing properties of CSs to a different degree in STs and GTs. In most of our studies to date, the lever or light CSs used could not only be approached, but in the case of a lever CS, it could also be physically engaged and manipulated. Several features of the CS, including its stimulus modality, whether it includes motion, the CS-US interval, spatial arrangement, and localizability influence the form of CRs [1], [25]–[37]. Therefore, as a first step, we were interested in determining whether individual variation in the extent to which a lever CS acquires incentive salience predicts the extent to which a simple auditory CS acquires incentive salience. To this end, we tested the ability of lever and auditory CSs to attract and/or to act as conditioned reinforcers in STs and GTs. In addition, it is known that multiple classes of drugs (e.g., amphetamine) and stressors can potentiate the conditioned reinforcing effects of reward cues [38]–[44]. However, these reinforcement-enhancing effects of amphetamine may depend on the modality of the CS, as well as the initial incentive value of the CS. Therefore, we also asked whether the ability of amphetamine or stress to potentiate conditioned reinforcement depends on the nature of the CS (i.e., lever vs. auditory CSs).

## Methods and Materials

### Ethics statement

All experiments followed the principles of laboratory animals care specified by “Guidelines for the Care and Use of Mammals in Neuroscience and Behavioral Research” National Research Council (2003), and all procedures were approved by the University Committee on the Use and Care of Animals at the University of Michigan (UCUCA# PRO00004467).

An overview of the phases within each of the experiments described here is provided in Table 1. In general, all experiments consisted of a pretraining phase (1–2 days), a PCA phase (5 days), followed by additional conditioning phases and then a test for conditioned reinforcement. The details of each individual experiment are described below.

**Table 1:** General timeline of experimental phases.

Exp 1: Auditory CS	Pretraining (2 d)	PCA (5 d)	Habituation (2 d)	Tone Cond. (5 d)	Cond. Reinf. (1 d)
Exp 2: Compound CS	Pretraining (2 d)	PCA (5 d)	Cond. Reinf. (2 d)		
Exp 3: Sequential CSs	Pretraining (2 d)	PCA (5 d)	Habituation (2 d)	Tone Cond. (5 d)	Cond. Reinf. (2 d)
Exp 4: Amphetamine and Auditory CSs	Pretraining (1 d)	PCA (5 d)	Habituation (2 d)	Tone Cond. (5 d)	Cond. Reinf. (1 d)
Exp 5: Amphetamine and Lever CSs	Pretraining (1 d)	PCA (5 d)	Cond. Reinf. (1 d)		
Exp 6: Stress	Pretraining (1 d)	PCA (5 d)	Self-admin. (12 d)	Extinction (7–9 d)	Cond. Reinf. (1 d)

### Experiment 1

#### Conditioned reinforcing effects of an auditory CS in STs and GTs

The purpose of this experiment was to determine whether variation in attributing incentive salience to a lever CS, assessed using PCA, predicts variation in attributing incentive salience to an auditory CS. Cleland and Davey [27] demonstrated that even if rats can localize an auditory stimulus, they will not readily approach it in Pavlovian setting. Therefore, in rats, approach behavior cannot be used to assess the extent to which an auditory CS acquired incentive salience, which is why in this experiment we used a test of conditioned reinforcement. To this end, rats first underwent PCA training to assess the attractiveness of a lever CS (i.e., sign tracking vs. goal tracking), and then in a subsequent phase they were trained using a tone CS. Following this conditioning, rats were given a conditioned reinforcement test to assess the incentive value of the tone CS in STs and GTs.

#### Subjects

Forty-seven male Sprague-Dawley rats (200–250 g upon arrival) were purchased from Harlan (Haslett, IN) and housed in groups of 2–3 for one week prior to testing, during which time they were gently handled daily. Experimental testing occurred during the dark phase of a 12∶12 h reverse light/dark cycle (Lights off at 8 am). Food and water were available ad-libitium except during testing sessions. Rats were given ∼25 banana-flavored pellets (Bioserve #F0059) once daily for two days preceding the first test session. They were not food deprived between testing sessions in any experiment.

#### Apparatus

Rats were tested in Med-Associates conditioning chambers (20.5×24.1 cm floor area, 29.2 cm high; Med-Associates Inc., St. Albans, VT) that were placed inside sound-attenuating cubicles equipped with ventilation fans. Inside the chambers, a retractable lever with an LED backlight was placed on the left or right side of a food magazine, into which the banana-flavored food pellets were delivered using an automated feeder. A red house light was located opposite the feeder near the top of the chamber. Lever deflections and magazine entries (photobeam breaks) were recorded using Med-PC IV software. For auditory conditioning sessions, a 2.9 kHz tone was delivered through speakers mounted near the ceiling of the chamber. For conditioned reinforcement sessions, the food magazine was removed, and the lever or tone was placed in between two nose-poke ports.

#### Pretraining (2 days)

Rats received two pretraining sessions (once per day for two days), during which 50 banana-flavored pellets were delivered into the food magazine on a VI 30 (0–60 s) after an initial 5 min habituation period.

#### Pavlovian conditioned approach (PCA) sessions (5 days)

After the pretraining, rats were subjected to 5 daily PCA (‘autoshaping’) training sessions, consisting of 25 trials/day of lever-pellet pairings. Trials were separated by 30–150 s (i.e., a VI90 schedule). For each trial, an illuminated lever was extended into the conditioning chamber for 8 s and immediately upon lever retraction a banana-flavored food pellet was delivered into the food magazine. Lever deflections and magazine entries were recorded during the CS and intertrial intervals (see [3], [9]). Note that rats were not food deprived at any time.

Data from PCA sessions were used to classify rats as sign-trackers (ST), goal-trackers (GT), or intermediates (IN) based on a “PCA Index” derived from a recent meta-analysis of a large sample of heterogeneous rats [3]. Briefly, the PCA Index is a score from −1.0 to 1.0, calculated as the average of (a) response bias [(number of lever presses − number of magazine entries during the CS)/(number of lever presses + number of magazine entries during the CS)], (b) approach probability difference [(number of trials with at least one lever press − number of trials with at least one magazine entry during the CS)/25], and (c) latency difference (latency to approach magazine during the CS − latency to approach lever)/[8]). We operationally defined STs as rats with an average PCA score from 0.4 to 1.0 for the last 2 days of conditioning and GTs as rats with a score of −0.4 to −1.0. INs had scores ranging from 0.39 to −0.39.

#### Context extinction sessions (2 days)

Relative to STs, GTs spend more time near the food magazine during conditioning [3]. Since this would produce short food- magazine approach latencies and might enhance their ability to learn future tone-pellet associations, rats underwent two habituation sessions in which rats were placed into the conditioning chamber for 40 min with the red houselight illuminated. By promoting exploration of the chamber, these sessions circumvented potential spurious group differences during the subsequent auditory conditioning sessions.

#### Auditory conditioning (5 days)

Following the context extinction sessions, rats were trained during five daily sessions of 25 tone-pellet trials/day delivered on a VI 90 schedule. For each trial, the 2.9 kHz tone was presented for 8 s preceding the food pellet delivery. Magazine entries during the CS and intertrial intervals were measured.

#### Conditioned Reinforcement (1 day)

For conditioned reinforcement test sessions, the food magazine and retractable levers were removed from the chambers. Nose poke ports equipped with photobeam detectors were inserted to the left and right of where the magazine was previously located. Rats were placed into the chamber for a 5-min habituation period, after which time the houselight was illuminated, and rats were allowed to nose-poke for a 3 s presentation of the tone CS. Left or right nose-poke ports were designated as “active” (reinforced) or “inactive” on a counterbalanced basis. Nose-pokes into the active port were reinforced on an FR1 schedule, and the number of responses following (and including) the first reinforced response into the active hole were measured. Responses into the inactive hole were included to ensure that responding was maintained (reinforced) by the lever, and to assess potential non-specific changes in activity levels. The session lasted 40 min.

#### Statistics

The dependent variables for the PCA and auditory conditioning trials were lever deflections (PCA only) and magazine entries. Repeated-measures ANOVA was used to determine the effect of Phenotype (ST, GT, IN) and Day (1–5) on these measures. For conditioned reinforcement tests, the dependent variable was nose-poke port entries, and ANOVA was used to measure the effect of Port (Active vs. Inactive) as the within-groups factor and Phenotype as the between-groups factor. Fisher's LSD post-hoc tests were conducted to investigate significant main effects and interactions.

#### Note

In this and subsequent experiments, we did not include additional unpaired control groups for two reasons (besides the desire to reduce the number of animals used). 1) Our primary aim was to compare incentive salience attribution in STs and GTs, which does not require a comparison of paired vs. unpaired groups. 2) In numerous previous studies, we, and others, have included unpaired control groups and, using the same procedures as used here, have repeatedly shown that an Unpaired CS does not acquire incentive salience – that is, the CS and US must be paired [12], [17]–[19], [45]–[48].

### Experiment 2

#### Conditioned reinforcing effects of components of a compound stimulus

The aim of experiment 2 was to determine whether two components of a compound lever-tone CS would acquire incentive salience differentially in STs and GTs, as assessed by their ability to later alone act as conditioned reinforcers. We hypothesized that, if the tone and lever CSs acquired incentive salience through similar behavioral mechanisms, presentation of the lever CS would overshadow the attribution of incentive salience to the tone CS to a greater degree in STs, compared to GTs.

For this experiment, 47 male Sprague Dawley rats were tested as in Experiment 1 with the following exceptions: during the PCA phase of this experiment, the tone and the lever were presented simultaneously as a compound stimulus. This was followed by two conditioned reinforcement sessions for each rat (one per day). For the first session nose-pokes into the active port were reinforced by a 3 s tone presentation. For the second session, a 3 s presentation of the illuminated lever was the reinforcer.

### Experiment 3

#### Conditioned reinforcing efficacy of sequential auditory stimuli

The aim of experiment 3 was to address the possibility that STs and GTs are attending to different temporal aspects of sequential CSs. For example, GTs may be sensitive to stimuli located more closely in time to reward delivery (such as the sound associated with retraction of the lever). If this was the case, GTs would not be able to sign-track because their CS would only be present for a short period of time. Thus, we hypothesized that if GTs preferentially associate proximal stimuli with the US, (i.e., those stimuli occurring closer in time relative to the US), while STs associate distal stimuli with the US, (i.e., those stimuli occurring further away in time relative to the US), then proximal stimuli would be more reinforcing for GTs than STs. To test this hypothesis, we presented STs and GTs with two distinct auditory CSs (tone and white noise) presented in sequence.

The pretraining and PCA portions of this experiment were the same as in experiment 1, although INs were not tested further. Rats were then given 5 auditory conditioning sessions where a “distal” stimulus was presented for 2 s, followed by a 2 s pause, followed by the “proximal” stimulus for 2 s and another 2 s pause, after which the food pellet was delivered. The identity of the distal and proximal CSs (whether they were tones or white noise bursts) was counterbalanced. Magazine entries during each CS and the following 2 s pause were measured. On the two subsequent conditioned reinforcement test days, rats nose-poked for either the distal or the proximal stimulus. Only one CS (either the distal or the proximal CS) was the reinforcer during each of the two counterbalanced conditioned reinforcement test sessions. Interval (proximal, distal) was added to the ANOVA as a within-groups factor. As in experiments 1 and 2, Phenotype (ST, GT) and Port (Active, Inactive) were included as between- and within-groups factors, respectively.

### Experiment 4

#### The effect of amphetamine on the conditioned reinforcing effects of a tone CS

In the experiments above, we established that the extent to which stimuli associated with a food reward acquire incentive salience varies depending on the individual and the nature of the CS. Here we asked whether there is similar individual variation in the ability of amphetamine to *potentiate* the conditioned reinforcing effects of an auditory CS.

For experiments 4 and 5, rats were purchased from Harlan (Indianapolis, IN) or Charles River (Portage, MI). The procedures for PCA training were the same as in Exp. 1, with the following exceptions. (1) Magazine pretraining was reduced to 1 day of 25 pellets. We found that this reduced the number of overall magazine entries (during the CS and intertrial intervals) during the initial conditioning sessions. (2) Ten min before the conditioned reinforcement test, *independent groups* of rats were given an i.p. injection of 0.9% saline vehicle or one of three doses of amphetamine (0.25, 0.75, 2 mg/kg, weight of the salt; purchased from Sigma-Aldrich, St. Louis, MO). Data from INs were not included in the conditioned reinforcement analyses. ANOVAs were used to analyze effects of Phenotype (ST, GT) and amphetamine Dose (0, 0.25, or 0.75), with Day and Port (Active, Inactive) as the within-subjects measures.

### Experiment 5

#### The effect of amphetamine on the conditioned reinforcing effects of a lever CS

The purpose of experiment 5 was the same as Exp. 4, but to determine the effects of amphetamine on the conditioned reinforcing effects of a lever CS. In this experiment, rats were screened with PCA using lever-pellet presentations, after which they were given a test for conditioned reinforcement, in which nose-pokes into the active port were reinforced by 3 s lever presentations. Ten minutes before the conditioned reinforcement test, independent groups of rats were given an injection of saline or 0.25 or 0.75 mg/kg amphetamine. Data from INs were not included in the conditioned reinforcement analyses.

### Experiment 6

#### The effect of stress on the conditioned reinforcing effects of a drug CS

Experiments 4 and 5 established that amphetamine potentiated the conditioned reinforcing effects of both a tone CS and a lever CS to the same extent in STs and GTs, even when the lever CS initially acquired greater incentive salience in STs than GT. This suggests that the extent to which a cue acquires incentive salience does not predict the degree to which amphetamine can potentiate the motivational value of the same CS. We were interested in whether this would also be true using a different agent to potentiate conditioned reinforcement, and when the US was a drug rather than food. We asked, therefore, whether stress, like amphetamine, would enhance conditioned reinforcement to the same extent in STs than GTs.

The subjects were twenty male Sprague-Dawley rats (200–250 g) purchased from Charles River (Portage, MI). The rats underwent Pavlovian approach (autoshaping) training and the PCA score was used to identify STs (N = 9) and GTs (N = 11) as described above (INs were not used). The rats were then anesthetized with ketamine hydrochloride (100 mg/kg i.p.) and xylazine (10 mg/kg i.p.) and intravenous catheters were surgically implanted using a procedure described previously (Crombag et al, 2000).

#### Self-administration training

Seven days after surgery all animals were trained to self-administer cocaine using a procedure to insure that all animals took exactly the same amount of drug and received the same number of drug-CS pairings. Cocaine was used in this experiment because of its relatively short half-life compared to amphetamine, which allows for multiple cue-drug exposures in a shorter period of time. At the beginning of each training session, catheters were flushed with 0.2 ml saline, and animals were then placed in the testing chambers and connected to a swivel. Nose pokes into an active port resulted in a 20 s presentation of the white cue light (which served as the drug CS) inside the nose poke port, and an intravenous infusion of cocaine hydrochloride (0.4 mg/kg per infusion in 50 ml delivered over 2.6 s) on a fixed ratio (FR) 1 schedule. After the cocaine infusion, a 20 s timeout period began, where nose pokes in the active port had no consequences. To control for the number of infusions each rat received per session, an infusion criterion (IC) was used throughout all cocaine self-administration sessions [20]. On days 1–3, the infusion criterion was set to 10 infusions. In other words, once animals self-administered ten infusions, the session ended. On days 4–6, the infusion criterion was set to 20 infusions. The final infusion criterion of 20 infusions was implemented for the last six days of self-administration training (days 7–12).

#### Extinction and Reinstatement

Following self-administration training, rats were left undisturbed in their home cages for 7–9 days. After this incubation period, rats began extinction training [49]. During two hour daily sessions, rats were placed in the self-administration chambers and connected to the swivel. The sessions were identical to self-administration sessions with the exception that nose pokes into the active port only resulted in presentation of the cue light (no drug infusion). The cue was extinguished to allow us to determine whether stress renews the incentive motivational properties of the cue. Animals were trained under extinction conditions for ten days (one session per day). On the last three days of extinction training rats given an i.p. injection of sterile water (vehicle) prior to the session to habituate them to this procedure. On the first reinstatement test day, which occurred the day immediately after extinction training, half the rats received an i.p. injection of the vehicle and half yohimbine (2.5 mg/kg) and they were then placed into the test chambers. A number of studies have reported that yohimbine, which induces a stress response, is effective in reinstating cocaine-seeking behavior [50], [51]. The reinstatement test session conditions were identical to those during extinction; that is, a nose poke resulted in presentation of the drug cue, but not the drug. The following day animals resumed extinction training until they again reached extinction criterion (less than 25 active responses in a session), and then the next day a second reinstatement test was conducted. This was identical to the first reinstatement test except animals that first received vehicle now received yohimbine and the other half the vehicle. Active and inactive nosepoke responses were recorded during extinction (pre-stress Session) and reinstatement (post-stress Session) tests and were used in data analysis.

## Results

### Experiment 1

**Figure 1:**
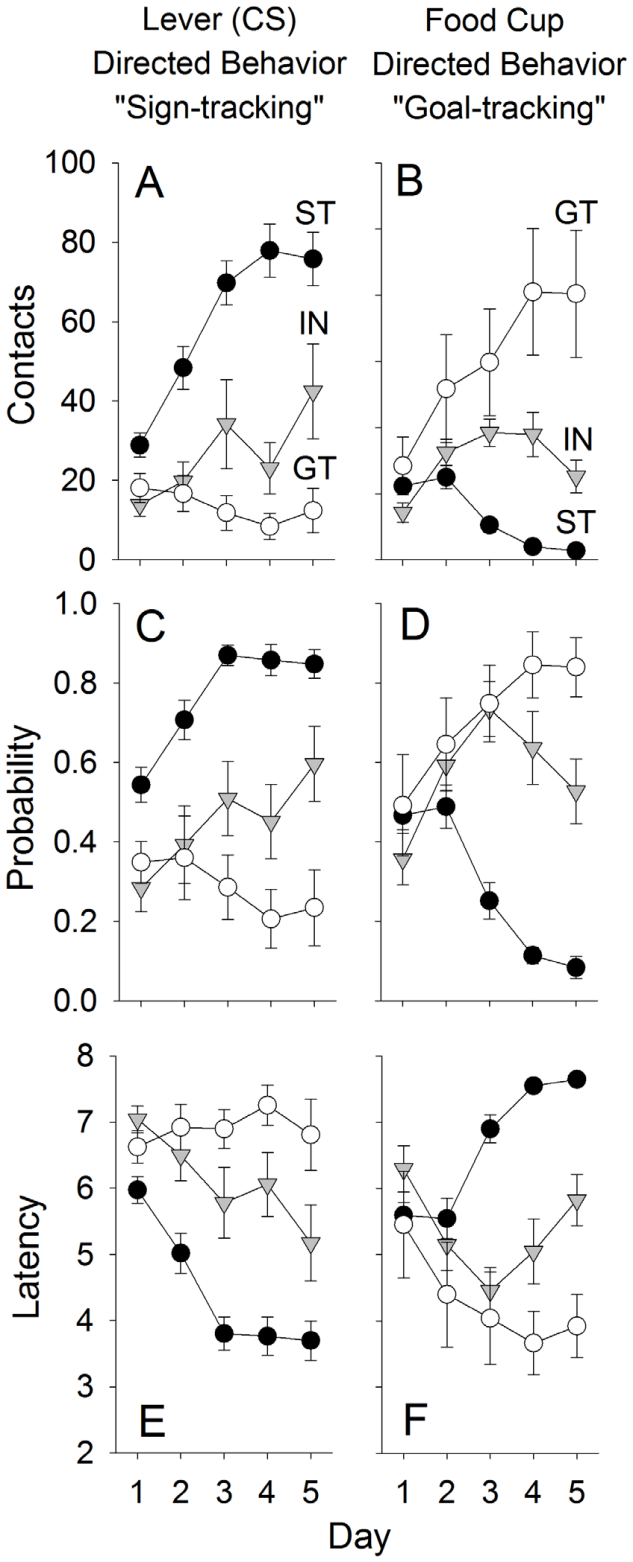
Pavlovian Conditioned Approach (PCA). Rats (n = 47) were classed as sign-trackers (ST), intermediates (IN) or goal-trackers (GT) based on CS-evoked behaviors during 5 days of Pavlovian training. Values represent mean (± SEM) number of lever deflections (panel A), food cup entries (panel B), probability of approaching the lever (panel C), or food cup (panel D), and latency to contact the lever (panel E) or make a food cup entry (panel F).

**Figure 2:**
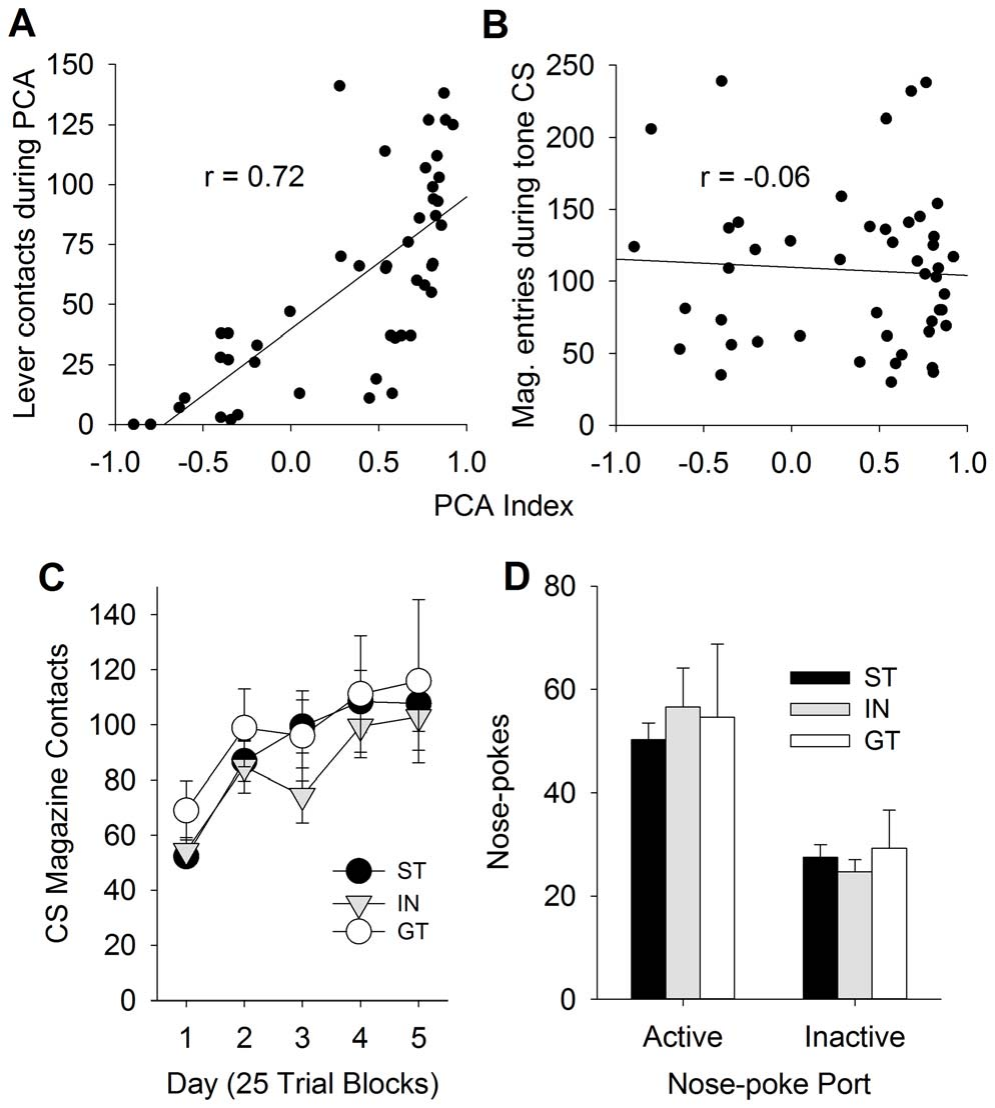
Pavlovian Conditioning using a tone CS. PCA Index scores were correlated with CS lever contacts during PCA (panel A) but not with CS magazine entries during the last day of tone conditioning (panel B). During tone conditioning (panel C), only goal-tracking was observed and did not differ between sign- and goal-trackers. When allowed to nose-poke for the tone in a conditioned reinforcement test (panel D), STs and GTs did not differ in the number of nose-pokes. Data are represented as mean (± SEM).

Of the 47 rats tested, 29 STs, 7 GTs, and 11 INs were identified using the PCA Index score described above. Fig. 1 shows that, with training, STs and GTs increasingly approached the lever or magazine, respectively, as indicated by changes in the probability, number of contacts, and approach latency over sessions (Fig. 1; Fs (8, 172)>3.6; ps<0.001 for the Phenotype by Day interaction for all six measures). These data are similar to many previous studies (for review see [5]). Fig. 2A shows that there was strong positive correlation (r = 0.72, p<0.05) between the PCA Index score and the number of lever deflections on day 5 of conditioning. This is expected, as the lever contacts are included as part of the PCA Index score.

When rats were conditioned using tone-pellet pairings, they all learned to approach the food-magazine (i.e., they all goal-tracked). This goal-tracking response increased over the five days of training [Fig. 2C; F (4, 176) = 12.5, p<0.001 for the main effect of Day], and there were no differences between STs and GTs (or INs) in the acquisition of the GT CR. Furthermore, there was no correlation between the PCA Index score and number of magazine entries elicited by the tone CS on the last day (day 5) of conditioning (Fig. 2B; r = −0.06), suggesting that approach to the lever CS and magazine entries evoked by the tone CS are not related. This is consistent with previous reports (e.g., [1], [5], [27]) that the nature of the CR is dependent on the type of CS, with an auditory CS eliciting only goal-tracking while a lever CS elicits either goal- or sign-tracking, depending on the individual, and other factors, such as reward uncertainty [52], [53].

Next, to determine if the tone CS acquired incentive stimulus properties differentially in STs and GTs, all rats were subjected to a test for conditioned reinforcement. Fig. 2D shows that rats made more nose-pokes for the tone CS than nose-pokes into the inactive port [F (1, 44) = 61.1, p<0.001 for the main effect of Port], and there were no group differences. That is, the tone CS was equally reinforcing in STs and GTs, suggesting that the acquired reinforcing efficacy of an auditory CS is dissociable from the attractive or reinforcing properties of a lever CS.

### Experiment 2

**Figure 3:**
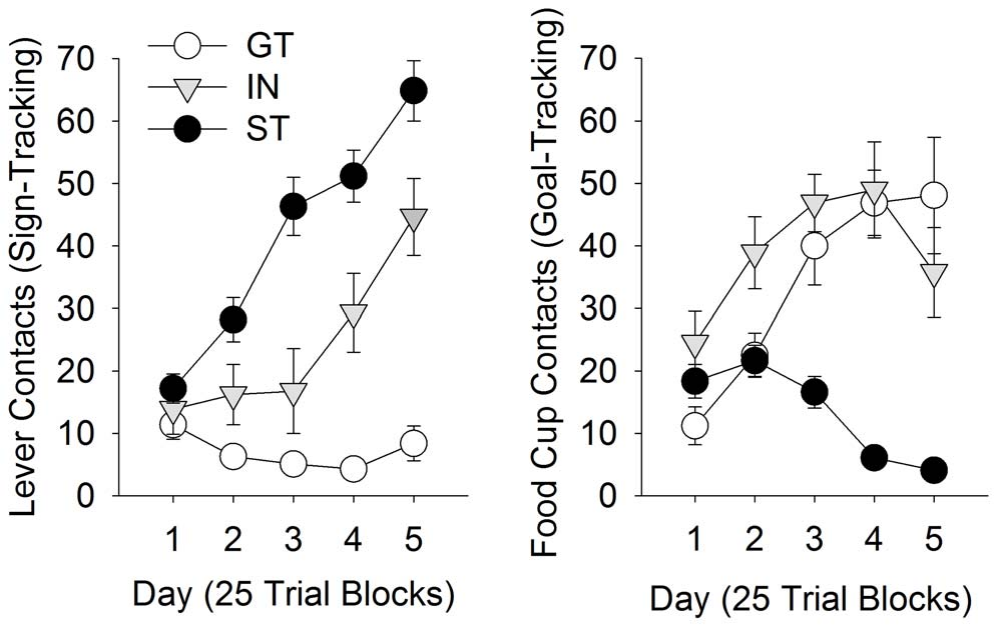
PCA behaviors using a compound CS. A) Sign-trackers (ST), goal-trackers (GTs), and intermediates (INs) were identified based on PCA using a compound tone/lever-cue. STs and GTs demonstrated either lever-directed (A) or goal-directed (B) behavior, respectively, during training.

Of the 47 rats tested, 24 STs, 9 GTs, and 14 INs were identified, based on the PCA Index scores. During training with the compound lever/tone stimulus, individual differences in the form of the CR (ST vs. GT) emerged, as in experiment 1. With training, some rats (STs) learned to approach the lever, and others (GTs) the food magazine (Fig. 3; Fs (8, 176)>7.7; ps<0.001 for the Phenotype by Day interaction for both measures). While not an explicit goal of this experiment, the similar PCA scores (mean: 0.34, SEM: 0.08) compared to experiment 1 (mean: 0.23, SEM: 0.08) suggest that presenting the tone in compound with the lever did not significantly alter PCA behavior [t (1, 97) = 0.98, p>0.05]. This result would be expected if the tone CS and lever CS acquired incentive salience through separate mechanisms. Otherwise, the auditory component of the CS would be expected to promote more goal-tracking in rats that would otherwise be sign-tracking, thereby reducing the number of rats identified as STs.

**Figure 4:**
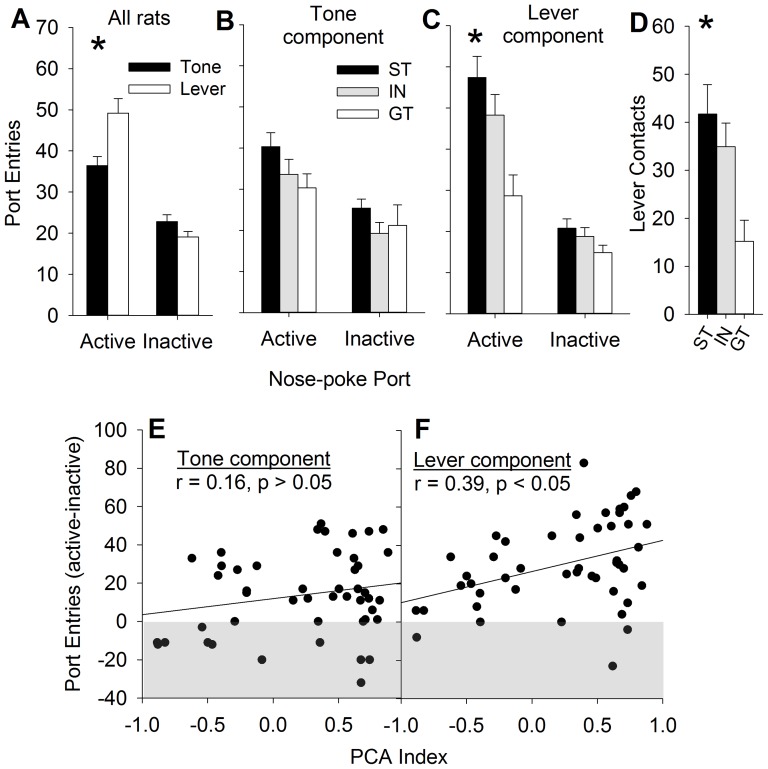
The lever, but not the auditory, component of a compound CS are differentially reinforcing in sign-trackers (ST), but not goal-trackers (GT). Rats nose-poked for either the lever component or the auditory component of the compound CS in separate conditioned reinforcement tests. STs, GTs, and intermediates (IN) did not differ in nose-pokes that were reinforced by the tone component of the CS (panel A), but STs made more nose-pokes for the lever component (panel B). STs approached the lever more often than GTs during the conditioned reinforcement test (Panel C). The PCA index was significantly correlated with the reinforcing efficacy of the lever component of the CS, but not the auditory component (Panels D and E). Asterisks indicate significant differences compared to goal-trackers (p<0.05). Data are represented as mean (± SEM).

Next, the acquired reinforcing efficacy of the tone vs. lever elements of the compound CS were assessed during separate conditioned reinforcement tests (Fig. 4A). Both the tone and lever components of the compound CS were effective reinforcers, but the lever was a more effective reinforcer than the tone (Fig. 4A). This may have been due to order and/or overshadowing effects [54], but nevertheless, it is consistent with a recent report that a lever CS is more effective conditioned reinforcer than an auditory CS, even when presented separately in the same training session [55].

However, the tone element was an equally effective conditioned reinforcer in STs and GTs (Fig. 4B; p>0.05). In contrast, the lever element was a significantly more effective conditioned reinforcer in STs (and INs) than GTs [Fig 4C; F (2, 44) = 3.9, p<0.05 for the Phenotype x Port interaction, followed by Fisher's post-hoc comparisons, ps<0.001]. In addition, STs and INs made significantly more active responses than GTs (p<0.01, Fisher's post-hoc comparison). As expected, STs also made more lever contacts during the brief period the lever was presented [Fig. 4D, F (2, 44) = 3.8, p<0.05 for the main effect of Phenotype, followed by Fisher's post-hoc comparisons p<0.05]. Finally, the reinforcing efficacy of the lever component of the CS, but not the auditory component, was significantly correlated with the PCA index scores (Figs. 4E and 4F; p<0.05). Together, these results demonstrate that the tendency to approach the lever during conditioning was associated with the reinforcing efficacy of the lever, but not the tone.

### Experiment 3

**Figure 5:**
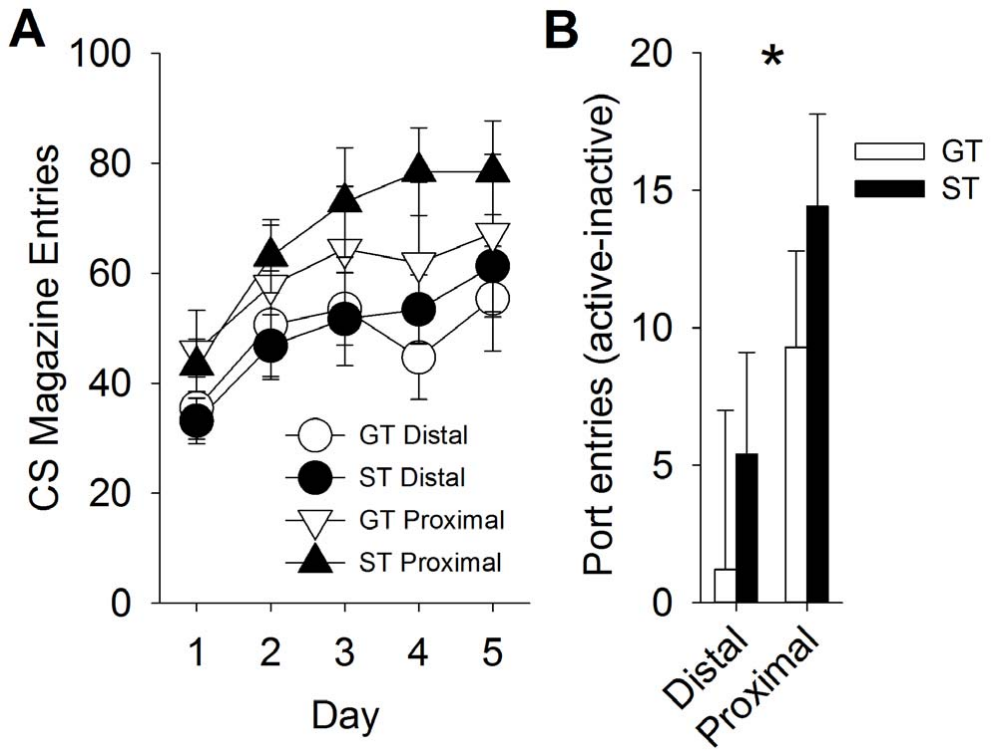
Proximal auditory stimuli (either tone or white noise) are more reinforcing than distal stimuli. Rats made more food-cup entries during the proximal stimulus presentation during training (panel A), and performed more nose-pokes for the proximal stimulus (panel B). Data are represented as mean (± SEM). There were no significant differences between sign-trackers (ST) and goal-trackers (GT).

Out of 42 rats tested during PCA, 16 STs, 10 GTs, and 16 INs were identified as described in Exp. 1. As expected, when sequential auditory CSs were paired with food-pellet delivery, rats entered the magazine more during the proximal CS compared to the distal CS [Fig 5A; F (1, 20) = 11.1, p<0.01 for the main effect of Interval]. There were no significant differences between STs and GTs (p>0.05). This demonstrates that rats enter the magazine more during the proximal stimulus than the distal stimulus. This is probably because rats had already approached and entered the food magazine by the time the proximal stimulus was presented. Therefore, the important tests of whether the distal and proximal CSs differentially acquired motivational properties are the conditioned reinforcement tests, which were given subsequent to the Pavlovian conditioning sessions. For the conditioned reinforcement test, rats responded significantly more for the proximal stimulus, relative to the distal stimulus [Fig 5B; F = (1, 20); 9.0, p<0.01 for the main effect of Interval], which indicates that the proximal stimulus was more reinforcing. Compared to GTs, STs had elevated responding over all [F (1,20) = 9.2; p<0.01 for the main effect of Phenotype]. However, this effect did not interact with Interval (p>0.05), which indicates that that STs did not respond more for the distal or proximal CSs than GTs. Thus, it does not appear that STs and GTs make associations between different temporal components of an auditory CS and US.

### Experiment 4

**Figure 6:**
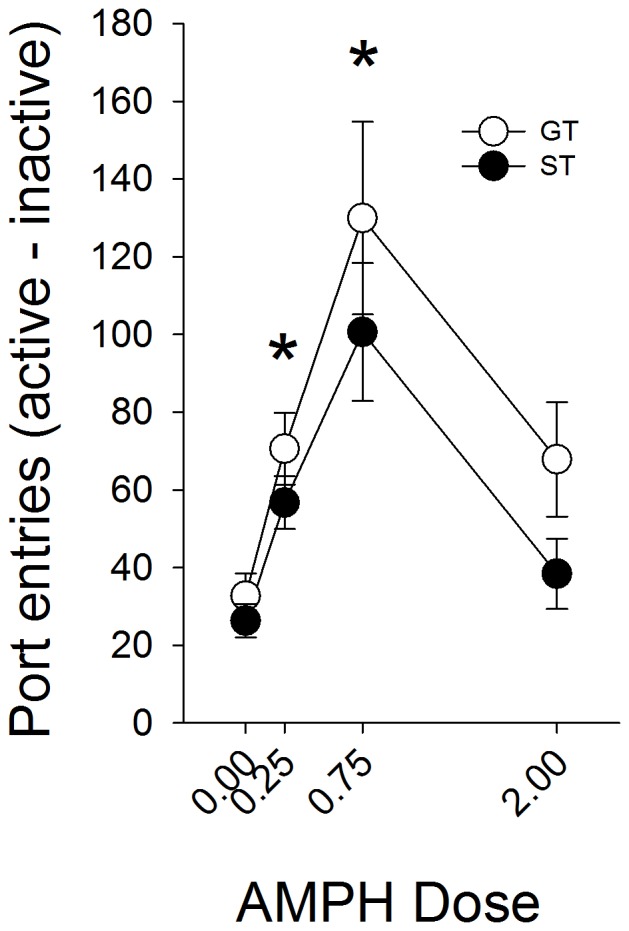
Amphetamine enhances the reinforcing efficacy of an auditory stimulus equally in sign-trackers (ST), goal-trackers (GT), and intermediates (IN). Amphetamine increased the number of nose-pokes into the active (reinforced by the tone-cue) port. Amphetamine did not have systematic effects on nose-poke responding into the inactive port. Data are represented as mean (± SEM). There were no significant differences between STs and GTs. Asterisks denotes a significant increase compared to the saline-treated rats (0.00 dose).

Out of the 192 rats tested for PCA, 80 STs and 65 GTs were identified (data not shown). When administered before the conditioned reinforcement test, amphetamine dose-dependently enhanced the conditioned reinforcing effect of the food-paired tone [Fig. 6; F (3, 137) = 15.7, p<0.05 for the main effect of Dose]. However, there were no differences between STs and GTs (ps>0.05), indicating that amphetamine potentiated the conditioned reinforcing effects of the tone cue to the same extent in STs and GTs.

### Experiment 5

**Figure 7:**
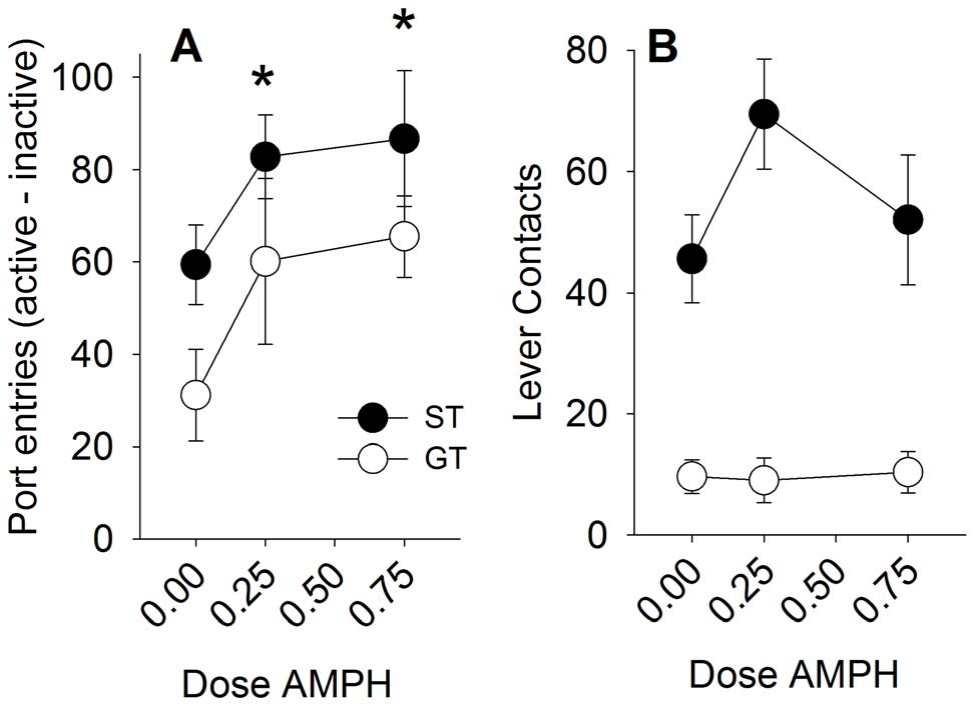
Amphetamine increases the reinforcing efficacy of the lever-cue equally in sign-trackers (ST), and goal-trackers (GT). Panel A demonstrates that STs responded more for the lever CS than GTs, and that amphetamine enhanced this conditioned reinforcement similarly in STs and GTs. Panel B shows that STs had more lever contacts than GTs, which was enhanced by the lower dose of amphetamine only in STs. Data are represented as mean (± SEM). There were no significant differences between STs and GTs. Asterisks denotes a significant increase compared to the saline-treated rats (0.00 dose).

Out of the 120 rats tested for PCA, 50 STs and 42 GTs were identified (data not shown). As in experiment 2, and in previous studies (e.g., Robinson and Flagel, 2009), under control conditions (following treatment with saline) the lever CS was more effective as a conditioned reinforcer in STs than GTs [Fig. 7A; F (2, 86) = 3.4, p<0.05 for the Phenotype x Port interaction, followed by Fisher's post-hoc test, p<0.05)]. Amphetamine potentiated the conditioned reinforcing effects of the lever CS [F (2, 86) = 4.0, p<0.05 for main effect of Dose], but there were no group differences in this effect of amphetamine (ps>0.05 for the interaction between Phenotype and Dose, Fig. 7A). As expected, during the conditioned reinforcement test, STs deflected the lever more than GTs, even though it was presented for only 3 sec [F (1, 86) = 60.2, p<0.001 for the main effect of Phenotype], but there were no statistically significant effects of amphetamine or interactions with Phenotype on lever contacts during the conditioned reinforcement test (ps>0.05).

### Experiment 6

The behavior of the rats during PCA training was essentially the same as described above for other experiments, so the data are not shown. There were no significant differences between STs and GTs in the acquisition of self-administration behavior across the twelve days of self-administration training, consistent with previous reports ([20], [21]; data not shown). By the last day of training, all animals discriminated between the active (ST mean active responses = 82.89, sem = 16.6; GT mean = 86.7, sem = 26.45) and inactive ports (ST mean inactive responses = 5.33, sem = 2.54; GT mean = 5.0, sem = 3.04). Across extinction training sessions both STs and GTs decreased responding to relatively low levels, and there were no group differences in the rate of extinction and inactive responses (ps>0.05), again, consistent with our previous studies [20], [21].

**Figure 8:**
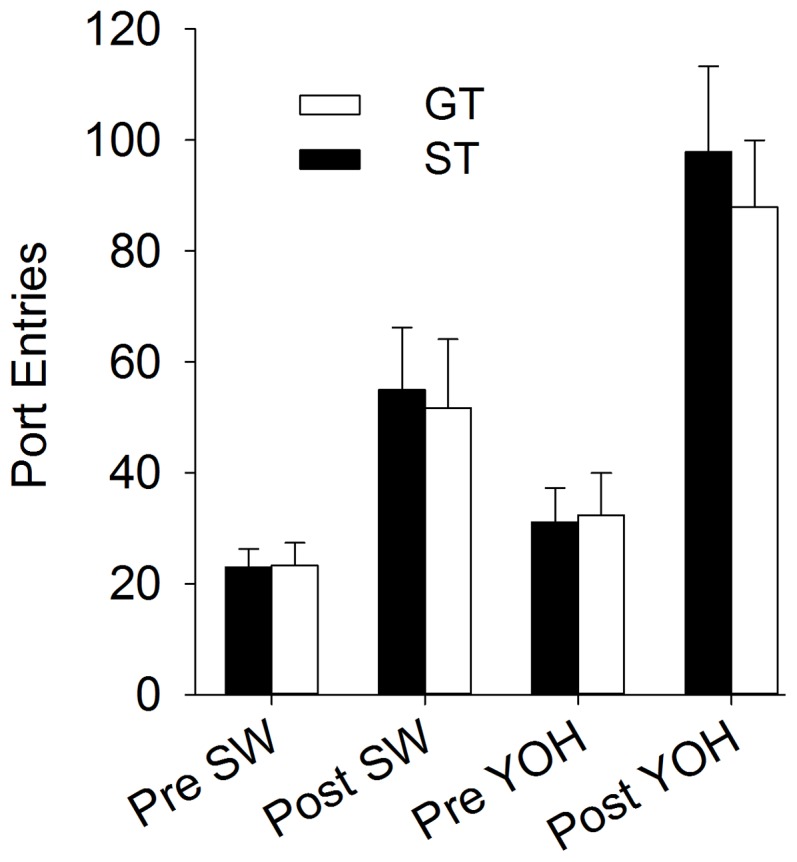
Stress potentiates the conditioned reinforcing properties of reward paired cues equally in sign trackers (ST) and goal trackers (GT). Nosepoke responses in sessions immediately before and after an ip injection of sterile water (SW) or the pharamacological stressor yohimbine (YOH). Responding after administration of yohimbine was significantly greater than responding after administration of sterile water, but increased equally in STs and GTs after both sterile water and yohimbine injections.

Fig. 8 shows the number of active responses on the reinstatement test day. A two-way ANOVA was used to analyze responding during the pre (extinction) and post (reinstatement) stress Sessions. Both STs and GTs showed significantly higher responding during the post stress period [F (1, 18) = 55.0; p<0.001 for the main effect of Session], but there were no differences between STs and GTs (ps>0.05). Interestingly, a vehicle injection also significantly reinstated responding [F (1,18) = 16.37; p = 0.001 for the main effect of Session], but again there were no differences between STs and GTs (ps>0.05). Of course, a vehicle injection is itself a mild stressor, which may explain why it produced a significant increase in responding. However, responding after yohimbine was significantly greater than after vehicle [F (1, 18) = 16.61; p = 0.001 for the main effect of Session]. Higher responding for yohimbine compared to vehicle cannot be explained by differences in baseline, as responding before both vehicle and yohimbine injection test days were not significantly different (p>0.05).

## Discussion

### Individual variation in the conditioned reinforcing effects of lever vs. auditory CSs

In a series of previous studies, we paired discrete, localizable and sometimes manipulable CSs (a lever or light) with the presentation of food or drug USs, and assessed the degree to which these CSs acquired incentive salience, as indicated by their ability to attract animals into close proximity to them, to act as conditioned reinforcers, or to produce conditioned motivation (e.g., to instigate reward seeking)[16], [20], [22], [23], [48]. We found that there is considerable variation in the extent to which individuals attribute incentive salience to such CSs – STs do so to a greater extent than GTs (see [3], [4] for reviews,[5]). Here we asked two questions. First, is there similar individual variation in the propensity to attribute incentive salience to an auditory CS, as assessed by its ability to act as a conditioned reinforcer? We replicated our previous work, showing that a lever CS acquired greater incentive value in STs than GTs. However, we also found that (1) overall, a tone-CS was a less effective conditioned reinforcer than a lever-CS; (2) when a tone was used as the CS all rats learned a GT CR, and the tone was an equally effective conditioned reinforcer in STs and GTs; and (3) when a compound lever-tone CS was used the tone element was an equally effective conditioned reinforcer in STs and GTs, but the lever component was a more effective conditioned reinforcer in STs. Second, we asked whether amphetamine or stress *potentiate* the conditioned reinforcing effects of different CSs in STs vs. GTs. We found that amphetamine potentiated the conditioned reinforcing effects of both a lever and tone CS to the same extent in STs and GTs. Similarly, yohimbine-induced stress renewed the reinforcing effects of a drug CS similarly in STs and GTs. Collectively, these results suggest that the properties of the CS itself influence the extent to which individuals attribute incentive salience to it, but not the ability of “incentive amplifiers [56]” to potentiate this process.

These findings cannot be due solely to overshadowing of the tone CS by the lever CS (see [54], [57]), the localizability of these CSs, or the temporal relationship to the US. First, no ST/GT differences in the reinforcing efficacy of the tone CS were observed whether the lever and tone CSs were presented in separate training sessions (Exps. 1, 3), or as a compound (Exp. 2). Second, the GT CR evoked by an auditory CS is unlikely due to the inability to localize the CS nor to a failure of the CS to acquire incentive value, because auditory CSs can elicit approach during instrumental paradigms [27]. Third, consistent with studies using other conditioning paradigms [28], [58], [59], we found that a proximal auditory stimulus was indeed a more effective conditioned reinforcer than a more distal stimulus. Whereas this suggests proximal CSs acquire greater incentive value, this effect was not different between STs and GTs. The most parsimonious account of these findings is that the nature of the approach response depends on the form of the stimulus, and the ability of an auditory CS to acquire reinforcing efficacy does not depend on whether it is approached. Future studies will be needed to determine to what extent the movement and manipulable [37] components of the lever CS contribute to these differences.

One interesting implication of these results is that what appears to be the *same* CR may be mediated by different psychological and neurobiological mechanisms depending on the CS that elicited it. For example, neither the acquisition nor expression of GT CR evoked by a lever CS is dopamine-dependent (at least in the core of the accumbens in the case of expression), although a ST CR is ([19], [60], [61],also see [62]). However, the existing literature suggests that a GT CR evoked by an auditory CS is dopamine-dependent ([63]–[65],see also [66], [67]). This suggests, of course, that the psychological processes that mediates learning a goal-tracking response (anticipatory head entries into a food cup) may differ depending on the properties of the CS that evokes it (see [3] for discussion,also see [54], [55]).

### Amphetamine and stress increase the incentive properties regardless of CS modality

Drugs can influence motivated behavior in multiple ways. For example, as argued by Caggiula and others [56], [68]–[71], the reinforcing properties of nicotine are due to a combination of three actions: the ability of the drug to act as a primary reinforcer, the ability of the drug to establish CSs as conditioned reinforcers through Pavlovian associations, and the ability of the drug to act “as a reinforcement enhancer, thereby magnifying the incentive value of accompanying stimuli, even if they are conditioned or unconditioned reinforcers.” Similar findings have been reported with other drugs [38], [72]–[74], which suggest that one behavioral mechanism of CS control over drug-taking behavior is the enhancement of the conditioned reinforcing properties of a CS by the drug. Here we show that amphetamine enhances reinforcing efficacy regardless of the cues' initial incentive value. This broad effect on behavior may be unique to psychostimulants compared to other drugs, and may be related to its ability to enhance the reinforcing efficacy of a broad class of cues [40], [74]–[81]. For example, the ability of nicotine to enhance the reinforcing effect of visual stimuli was systematically related to the strength of the reinforcer [82]. Our results suggest this is not the case for amphetamine. In addition, amphetamine has been reported to enhance the reinforcing efficacies of novel as well as unpaired stimuli, suggesting that pairing with a food reward is unnecessary for this reinforcement-enhancing effect [83], [84].

We also demonstrate here that a stressor (either an i.p. injection of vehicle or yohimbine) increased responding for a conditioned reinforcer to the same extent in STs and GTs. Although in this experiment we did not independently test for the conditioned reinforcing effects of the cocaine cue in the absence of the stressors, there is a wealth of evidence that cues that accompany self-administered cocaine acquire potent conditioned motivational properties [20], [47], [85], [86]. Thus, yohimbine appears to have similar broad effects on conditioned reinforcement, otherwise it would have been expected that the effect of yohimbine would be stronger in STs compared to GTs. This effect may be mediated by stress-induced increases in dopamine [51], [87], [88]. The ability of drugs and stress to increase incentive value may be particularly important for maintaining the influence of reward cues over motivated behavior.

### Implications for theories of Pavlovian conditioning

Theories of Pavlovian learning have largely focused on the associative structures involved in learning CS-US relations, but much less on how such learning actually influences behavior, i.e., on developing “performance models” of learning [12], [89]. Individual variation in the form of Pavlovian CRs, as described here, and by others (e.g., [12]), and the fact that this variation itself depends on the nature of the CS, emphasize the importance of developing performance models of learning [89]–[93]. Even CRs that appear the same in terms of their motor manifestation, as with a GT CR, may involve very different psychological and neurobiological processes depending on the properties of the CS that evokes it (see above), and one source of this variation is whether reward cues acquire incentive (or motivational) salience [3], [5], [18], [48]. These findings indicate that differences in the form of the CR are often not due to differences in associative strength, but rather, to the acquired incentive value of the CS. We suggest, therefore, that one factor any model of classical conditioning must incorporate, concerns the conditions under which CSs acquire incentive salience. This is not only of esoteric theoretical importance, but of practical importance as well. This is because conditions that promote incentive salience attribution to reward cues may also be the conditions that promote the ability of such cues to acquire an inordinate degree of control over behavior, leading to potentially maladaptive behavior – such as overeating in the case of food cues and pathological drug-seeking and drug-taking behavior in the case of drug cues [4], [5].
