# Correction: The Form of a Conditioned Stimulus Can Influence the Degree to Which It Acquires Incentive Motivational Properties

**DOI:** 10.1371/journal.pone.0107194

**Published:** 2014-08-26

**Authors:** The PLOS ONE Staff

The legend for Figure 4 is incorrect. The correct legend and the figure can be seen here.

**Figure 4:**
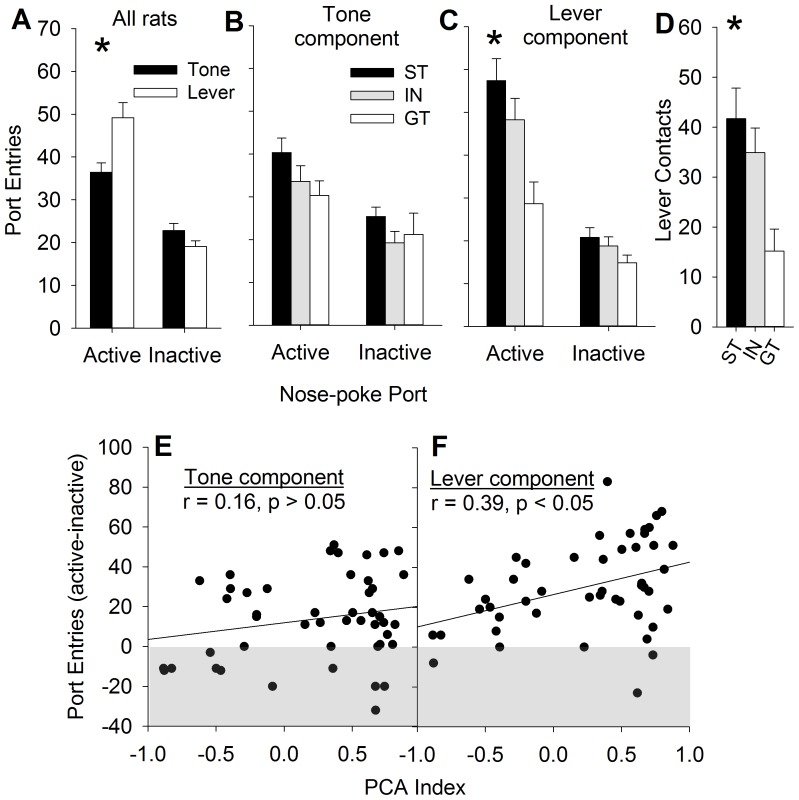
The lever, but not the auditory, component of a compound CS are differentially reinforcing in sign-trackers (ST), but not goal-trackers (GT). Rats nose-poked more for the lever component than the auditory component of the compound CS in separate conditioned reinforcement tests (Panel A). STs, GTs, and intermediates (IN) did not differ in nose-pokes that were reinforced by the tone component of the CS (panel B), but STs made more nose-pokes for the lever component (panel C). STs approached the lever more often than GTs during the conditioned reinforcement test (Panel D). The PCA index was significantly correlated with the reinforcing efficacy of the lever component of the CS, but not the auditory component (Panels E and F). Asterisks indicate significant differences compared to goal-trackers (p<0.05). Data are represented as mean (± SEM).
